# Impulsivity and consideration of future consequences as moderators of the association between emotional eating and body weight status

**DOI:** 10.1186/s12966-018-0721-1

**Published:** 2018-09-06

**Authors:** Marc Bénard, France Bellisle, Fabrice Etilé, Gérard Reach, Emmanuelle Kesse-Guyot, Serge Hercberg, Sandrine Péneau

**Affiliations:** 1 Equipe de Recherche en Epidémiologie Nutritionnelle, Centre de Recherche en Epidémiologie et Statistique Sorbonne Paris Cité, INSERM U1153, INRA U1125, Cnam, Université Paris 13, Bobigny, France; 2 Paris School of Economics and INRA, UMR1393 PjSE, 48 Boulevard Jourdan, 75014 Paris, France; 3 Service d’Endocrinologie, Diabétologie, Maladies Métaboliques, Hôpital Avicenne, Bobigny, France; 4 Département de Santé Publique, Hôpital Avicenne, Bobigny, France

**Keywords:** Emotional eating, Nutritional status, Consideration of future consequences, Impulsivity, Psychology

## Abstract

**Background:**

Emotional eating (EmE) is characterized by an over consumption of food in response to negative emotions and is associated with an increased weight status. Consideration of Future Consequences (CFC) or a low level of impulsivity could influence the association between EmE and weight status. The objective was to analyze the moderating influence of CFC and impulsivity on the relationship between EmE and BMI.

**Methods:**

A total of 9974 men and 39,797 women from the NutriNet-Santé cohort study completed the revised 21-item Three-Factor Eating Questionnaire to assess their EmE, the CFC questionnaire (CFC-12) to assess their level of time perspective, and the Barratt Impulsiveness Scale (BIS-11) to assess their impulsivity. Weight and height were self-reported each year over a median follow-up of 5.3 years. The associations between EmE and repeated measures of BMI were estimated by multiple linear mixed-effects regression models stratified by gender, tertiles of the CFC, or tertiles of the BIS-11, taking into account sociodemographic and lifestyle factors.

**Results:**

Overall, EmE was positively associated with BMI. CFC and impulsivity did not moderate the effect of EmE on changes of BMI per year, but quantitatively moderated the effect of EmE on overall BMI. In women, the strength of the association between EmE and weight status increased with CFC level. Difference of BMI slopes between a low and a high level of CFC was − 0.43 kg/m^2^ (95% CI: -0.55, − 0.30) (*p* < .0001). In addition, the strength of the association between emotional eating and weight status increased with impulsivity level. Difference of BMI slopes between a low and a high level of impulsivity was + 0.37 kg/m^2^ (95% CI: 0.24, 0.51) (*p* < .0001). In men, only individuals with a low CFC presented a stronger association of EmE with BMI.

**Conclusions:**

Impulsivity and consideration of future consequences moderated the association between emotional eating and body weight status. This study emphasizes the importance of taking into account psychological traits in obesity prevention.

**Electronic supplementary material:**

The online version of this article (10.1186/s12966-018-0721-1) contains supplementary material, which is available to authorized users.

## Background

Obesity represents one of the leading public health issues in many countries [1]. Behavioral traits, and in particular psychological traits, have been shown to influence food choices and BMI [2, 3]. More specifically, emotional eating (EmE), which is defined as eating in response to negative emotions was found to be positively associated with the intake of specific food groups such as high-fat and energy-dense snack foods [4–8] or sweet and fatty foods, although no association appeared with total energy or macronutrients [5, 9, 10]. Positive association of EmE with eating disorders has also been reported [11–15]. EmE has been repeatedly found to be associated with weight status [16–18], BMI [7, 19–22], and also with weight gain over a 2-year [23] and a 20-year [22, 24] follow-up periods. In addition, gender appeared to modify the relationship between EmE and weight status [21], and women generally report greater scores of EmE [5, 10, 21]. Finally, EmE appeared to fully mediate the relationship between personality factors such as neuroticism, low self-esteem and fear of intimacy, and weight loss following bariatric surgery and weight-loss program [25].

Some psychological constructs could be involved in the relationship between EmE and dietary habits or weight status by modulating the response to the urge to eat following negative emotions. Such could be the case of consideration of future consequences (CFC) and impulsivity which are important constructs relative to eating behavior and weight status. CFC corresponds to “the extent to which individuals consider the potential distant outcomes of their current behaviors and the extent by which they are influenced by these potential outcomes” [26]. Individuals with a high CFC are expected to adopt a future oriented behavior where long-term concerns are favored over immediate needs. In particular, individuals with a high CFC have been shown to exercise more [27–29], to be more sensitive to health communication [30], to have healthier eating attitudes and intentions [28], and lower BMI [31, 32]. Concern about the consequences of one’s behaviors could therefore influence responses to negative emotions. We therefore hypothesize that CFC could modify the association between EmE and weight status.

Impulsivity is a personality trait corresponding to “a predisposition toward rapid, unplanned reactions to internal or external stimuli without regard to the negative consequences of these reactions to the impulsive individual or to others” [33]. Impulsivity can be regarded as a consequence of impaired executive functioning [34]. Failures of the inhibitory processes lead to impulsive traits where urges to perform a specific act in response to a stimulus are not inhibited [34]. In the literature, impulsivity has been found associated with overeating [35–37], eating disorders [38–44], and weight status [3, 35, 45–49]. A low level of impulsivity and thus stronger inhibitory processes could provide the ability to inhibit and reduce urges to overeating in responses to negative emotions or regulate one’s dietary behavior regarding these impulses.

CFC has been shown to be inversely associated with impulsivity [32, 33] and both constructs are linked with measures of self-control [50–52]. However, CFC relies on intertemporal dilemmas and choices of one’s behavior [26], which could be seen as a proactive mechanism; whereas impulsivity would typically measure spontaneous responses and acts without forethought, which would involve less intervening cognitive and affective mechanisms [34].

The objective of this study was to assess how CFC and impulsivity moderate the relationship between EmE and repeated measures of BMI in a sample of the general population participating in the NutriNet-Santé cohort study, by taking into account sociodemographic and lifestyle characteristics.

## Methods

### Population

This study was conducted as part of the NutriNet-Santé study, which is a large ongoing web-based prospective cohort started in France in May 2009. The rationale, design and methods of the study have been described elsewhere [53]. Its overall aim is to explore the relationships between nutrition and health, and the determinants of eating behavior and nutritional status. Participants are adult volunteers (age ≥ 18 years) of the general French population with a scheduled follow-up of at least 10 years. At inclusion, participants have to complete several self-reported web-based questionnaires to assess their diet, their physical activity, anthropometric measures, lifestyle characteristics, socioeconomic conditions and health status. Participants complete this set of questionnaires every year after inclusion. Finally, another set of optional questionnaires related to determinants of eating behaviors, nutritional status, and specific aspects related to health are sent to every participant each month.

This study was conducted in accordance with the guidelines of the Declaration of Helsinki, and all procedures were approved by the International Research Board of the French Institute for Health and Medical Research (IRB Inserm n° 0000388FWA00005831) and the Commission Nationale Informatique et Libertés (CNIL n° 908,450 and n° 909,216). Electronic informed consent was obtained from all participants. The study was registered at clinicaltrial.org (Clinical Trial no. NCT03335644).

### Data collection

#### Emotional eating

Participants completed the French version of the revised 21-item Three-Factor Eating Questionnaire (TFEQ-R21) [54] 14 months after their inclusion (July 2010–January 2011 for most participants) and the completion of this questionnaire was optional. The questionnaire covers the following 3 aspects of eating behavior: cognitive restraint (6 items), emotional eating (6 items), and uncontrolled eating (9 items). The present analysis focused on the EmE scale that measures the propensity or need to overeat in response to negative feelings. EmE items are measured by using 4-point scales that ranged from “definitely true” (1 point) to “definitely false” (4 points). An example of the items of the EmE scale is as follows: *I start to eat when I feel anxious*. EmE score is calculated as a mean of all items within this dimension, so that the score ranges from 1 to 4. A score of 1 indicates an absence EmE, whereas a score of 4 indicates a high tendency to eat following negative emotions. Cronbach’s a coefficient showed excellent internal consistency of the EmE subscale in our sample (α = 0.93).

#### Consideration of future consequences

Consideration of Future Consequences was assessed with the French version of the CFC-12 questionnaire [55] completed from June to November 2014. The mean time interval from the administration of the TFEQ-R21 was 2.6 years (SD = 1.4). The CFC-12 is a 12-item self-report questionnaire [26] developed to measure the extent to which individuals consider distant versus immediate consequences of their behavior. Each item is measured on a 5-point Likert scale ranging from “extremely uncharacteristic” (1 point) to “extremely characteristic” (5 points). Examples of the items composing the CFC-12 are as follow: “I consider how things might be in the future, and try to influence those things with my day to day behavior” or “I only act to satisfy immediate concerns, figuring the future will take care of itself”. The total score is obtained by summing each item ratings leading to a possible range from 12 to 60 (higher scores indicating greater consideration of future consequences). A good internal consistency was obtained in our sample with a Cronbach’s α of 0.79. Participants were split into 3 categories determined by gender-specific tertiles of the total score: T1 (total score < 37 (women), < 38 (men)), T2 (37–44 (women), 38–44 (men)), and T3 (> 44 (women and men)).

#### Impulsivity

Impulsivity was assessed with the French version of the BIS-11 questionnaire [56] (derived from the French version of the BIS-10 questionnaire). The mean delay with the administration of the TFEQ-R21 was 2.6 years (SD = 1.4). The BIS-11 [57] is one of the most often used self-report questionnaire to assess impulsivity. It is a 30-item self-report questionnaire developed to assess the personality construct of impulsivity. Each item is measured on a 4-point Likert scale ranging from “rarely/never” (1 point) to “almost always/always” (4 points). Examples of the items composing the BIS-11 are as follow: “I plan tasks carefully” or “I do things without thinking”. The total score is obtained by summing all the item ratings, offering a possible range from 30 to 120, with higher scores indicating greater impulsivity. In our population, the BIS-11 displayed good internal consistency (Cronbach’s α = 0.78). Participants were split into 3 categories determined by gender-specific tertiles of the total score: T1 (total score < 55 (women) and < 54 (men)), T2 (55–62 (women), 54–61 (men) and T3 (> 62 (women), > 61 (men)).

#### Anthropometric measures

Self-reported height and weight data were collected each year using a web-based questionnaire. Repeated measures of BMI (kg/m^2^) were calculated as the ratio of weight to squared height. BMI data from the completion of the TFEQ-R21 (baseline BMI) to the last available data in the NutriNet-Santé cohort were used, representing up to 8 follow-up points per participant, covering a 7-year period. BMI change per year (kg.m^− 2^.y^− 1^) was calculated as BMI at the end of the follow-up minus baseline BMI divided by the duration of the follow-up.

#### Socio-demographic and lifestyle data

Potential confounders of the relationship between EmE and BMI were collected based on closest data to the date of completion of the TFEQ-R21: age (years), gender, education level (primary, secondary, undergraduate, and postgraduate), occupational status (unemployed, student, self-employed and farmer, employee and manual worker, intermediate profession, managerial staff and intellectual profession, and retired), monthly income, smoking status (never smokers and smokers), level of physical activity, and history of dieting (never dieters and dieters). More precisely, monthly income was calculated from information about income and composition of household. Members of a household were attributed different weights according to the OECD-modified equivalence scale: 1 for the first adult in the household, 0.5 for other persons aged 14 or older and 0.3 for children under 14 [58]. Total household income is then divided by the sum of the weights to yield a representative income. Categories of income were defined as followed: < 1200; 1200-1799; 1800-2299; 2300-2699; 2700-3699; and ≥ 3700 euros as well as “unwilling to answer”. Physical activity was assessed using a short form of the French version of the International Physical Activity Questionnaire (IPAQ) [59]. Weekly energy expenditure expressed in Metabolic Equivalent of Task (MET-minutes/week) was estimated and three levels of physical activity were constituted (low (< 30 min/day), moderate (30-60 min/day), and high (≥ 60 min/day)).

### Statistical analysis

A total of 51,394 participants (41%) of the NutriNet-Santé cohort study completed the CFC-12 and the BIS-11 questionnaires from the 125,377 subjects who received them. Among these participants 5399 did not complete the revised TFEQ-R21 questionnaire. From the 45,995 individuals left, 3002 women were excluded because they were pregnant at least once during the study, 461 individuals because of missing data on weight and height, 373 participants because they presented an acquiescence bias (agreeing to all questions without consideration of reversed items), and 2388 participants because they completed the TFEQ-R21 questionnaire after the CFC-12 and the BIS-11 questionnaires, leaving 39,771 participants in the final sample. Compared to excluded participants, the 39,771 participants in the final analysis were older (49.9 ± 13.7 years for included participants vs 41.8 ± 14.4 years for excluded participants, *p* < .0001), more often men (25.1% vs. 21.3%, *p* < .0001), and had a higher frequency of university education (33.2% vs. 30.7%, *p* < .0001).

The characteristics of the sample were compared by gender with Student’s t tests for continuous variables, and with Pearson’s chi-square tests for categorical variables. Since the distribution of the EmE score was not normal, analyses of differences in EmE were based on Wilcoxon two-sample and Kruskal-Wallis tests when appropriate. Longitudinal analyses of the association between EmE and repeated measures of BMI were performed with multiple linear mixed-effect regression models which were estimated with the maximum likelihood method. These models can deal with missing values on BMI (assumed to be missing at random) since they use all of the available data from an individual. First-order interaction test between EmE and gender was significant (*p* < .0001) and further analyses were stratified by gender. Second-order interactions were explored between EmE, time (as a continuous variable representing the delay between baseline BMI and follow-up points), and tertiles of CFC-12/BIS-11 to assess differences of BMI changes according to CFC/impulsivity levels. These interactions tests were all non-significant (CFC-12, men: *p* = .23/ CFC-12, women: *p* = .78/ BIS-11, men: *p* = .18/ BIS-11, women: *p* = .10). Therefore, interaction tests between EmE and tertiles of CFC-12/BIS-11 were carried out to look for the modification effect of these scales on BMI. These tests were all significant except for the test between EmE and tertiles of BIS-11 among men (CFC-12, men: *p* = .009/ CFC-12, women: p = <.0001/ BIS-11, men: *p* = .16/ BIS-11, women: *p* = <.0001). Final mixed models were accordingly stratified by gender and tertiles of the CFC-12 and tertiles of the BIS-11 and adjusted for time, age, education level, occupational status, monthly income per household unit, smoking status, physical activity, and history of dieting as fixed effects. The covariance structure of each model was selected according to the Akaike information criterion and the Bayesian information criterion. Intercept and time were included as random variables with a different variance component for each random effect among men models and a correlated random coefficient model (unstructured G matrix) among women. In addition, a spatial power law covariance structure of the R matrix was used to take into account unequally spaced longitudinal measurements in all models. Effect sizes were calculated by β and 95% confidence intervals (95% CI). Missing data on confounders were handled with multiple imputation by fully conditional specification (20 imputed datasets). All tests of statistical significance were 2-sided and significance was set at 5%. A Holm-Bonferroni procedure (step-down adjustment) was applied to correct for multiple testing. Statistical analyses were performed using SAS software (SAS Institute Inc., version 9.4).

## Results

### Description of the population

Table 1 shows characteristics of the sample according to gender. Compared with men, women were younger, fewer had a primary or secondary level of education, were less often retired, had a lower monthly household income, had a higher frequency of smoking, lower physical activity, and dieting, had a lower BMI at baseline (but a higher BMI change per year over the period of follow-up), and were less often overweight. In addition they had a lower level of CFC, a higher level of impulsivity, and a higher EmE score.

**Table 1:** Characteristics of 39,771 participants according to gender (NutriNet-Santé study, 2014)

	All (*n* = 39,771)	Women (*n* = 29,797)	Men (*n* = 9974)	P^1^
Age (years)^2^	49.9 ± 13.7	48.5 ± 13.5	54.1 ± 13.6	<.0001
Education level (%)				<.0001
Primary	2.8	2.7	3.3	
Secondary	32.8	32.4	34.3	
Some college	30.9	33.2	24.0	
University	33.2	31.5	38.2	
Missing data	0.2	0.2	0.2	
Occupational status (%)				<.0001
Unemployed	10.0	11.9	4.5	
Student	3.5	4.1	1.5	
Self-employed, farmer	1.7	1.6	2.0	
Employee, manual worker	16.0	18.5	8.5	
Intermediate professions	16.5	18.1	11.7	
Managerial staff, intellectual profession	22.1	20.6	26.7	
Retired	30.1	25.1	45.1	
Monthly household income (%)^3^				<.0001
< 1200€	13.3	14.6	9.3	
1200-1799€	23.1	23.7	21.4	
1800-2299€	15.3	15.4	14.7	
2300-2699€	9.5	8.7	11.8	
2700-3699€	16.5	15.3	20.0	
≥ 3700€	11.1	9.6	15.6	
Unwilling to answer	10.3	11.4	6.8	
Missing data	1.0	1.2	0.4	
Smoking status (%)				<.0001
Never smokers	48.7	51.6	40.0	
Smokers	51.3	48.4	60.0	
Physical activity (%)				<.0001
Low	21.6	22.3	19.4	
Moderate	40.5	42.1	35.6	
High	34.4	31.9	42.0	
Missing data	3.5	3.7	3.0	
History of dieting (%)				<.0001
Never dieters	60.8	57.7	70.3	
Dieters	39.2	42.3	29.7	
Baseline BMI (kg/m^2^)^2^	24.0 ± 4.5	23.6 ± 4.6	25.1 ± 3.8	<.0001
BMI change/year (kg.m^−2^.y^−1^)^2,4^	0.04 ± 0.48	0.05 ± 0.51	0.02 ± 0.36	<.0001
Period of follow-up (years)^4,5^	5.27 (4.00–6.93)	5.19 (3.97–6.91)	5.96 (4.00–6.94)	<.0001
Baseline Weight status (%)				<.0001
Underweight (< 18.5 kg/m^2^)	4.7	5.9	1.1	
Normal (≥ 18.5 and < 25 kg/m^2^)	63.4	66.4	54.1	
Overweight (≥ 25 and < 30 kg/m^2^)	22.9	18.6	35.5	
Obese (≥ 30 kg/m^2^)	8.9	8.8	9.3	
Missing data	0.1	0.1	0.0	
CFC (CFC-12) (range: 12–60)^2^	40.4 ± 7.0	40.2 ± 7.0	40.8 ± 6.9	<.0001
Impulsivity (BIS-11) (range: 30–120)^2^	58.6 ± 8.0	58.9 ± 7.9	57.4 ± 8.0	<.0001
Emotional eating score (range: 1–4)^5^	2.00 (1.33–2.83)	2.17 (1.67–2.83)	1.67 (1.00–2.17)	<.0001

*CFC-12* Consideration of Future Consequences scale, high CFC scores indicate a high level of future orientation

*BIS-11* Barratt Impulsiveness Scale (11th version), high BIS-11 scores indicate a high level of impulsivity

^1^*p*-value based on t tests for continuous variables or chi-square tests for categorical variables (corrected for multiple testing with a Holm-Bonferroni procedure)

^2^Mean ± SD

^3^Members of a household received different weights according to the OECD-modified equivalence scale: 1 for the first adult in the household, 0.5 for other persons aged 14 or older and 0.3 for children under 14 [58]. Total household income is then divided by the sum of the weights s to yield a representative income

^4^Based on the 38,665 participants with at least two measures of BMI

^5^Median (Interquartile range), adjusted p-value based on Wilcoxon two-sample test

### Relationship between EmE, CFC and impulsivity

Table 2 presents means of EmE according to levels of CFC and impulsivity by gender. Women with a high level of CFC had a lower score of EmE, although differences were relatively small. No significant differences were found for men. Women and men with a high level of impulsivity had a higher score of EmE. CFC and EmE were significantly correlated in our study (*r* = − 0.04, *p* < .0001 in women and *r* = − 0.02, *p* = .04 in men). Impulsivity and EmE were also significantly correlated (*r* = 0.16, *p* < .0001 in women and *r* = 0.17, *p* < .0001 in men). Overall, CFC was negatively associated with impulsivity (*r* = − 0.40 in women, *p* < .0001 and *r* = − 0.43, *p* = <.0001 in men).

**Table 2:** Consideration of future consequences (CFC), impulsivity, and emotional eating scores in 39,771 participants (NutriNet-Santé study, 2014)

GENDER	CFC	Emotional eating score		Impulsivity	Emotional eating score	
		Median (Interquartile range)	P^1^		Median (Interquartile range)	P^1^
WOMEN	CFC Tertile 1	2.33 (1.67–3.00)	<.0001	Impulsivity Tertile 1	2.00 (1.33–2.67)	<.0001
	CFC Tertile 2	2.17 (1.67–2.83)		Impulsivity Tertile 2	2.17 (1.67–2.83)	
	CFC Tertile 3	2.17 (1.50–2.83)		Impulsivity Tertile 3	2.33 (1.83–3.00)	
MEN	CFC Tertile 1	1.67 (1.00–2.17)	.78	Impulsivity Tertile 1	1.50 (1.00–2.00)	<.0001
	CFC Tertile 2	1.67 (1.17–2.17)		Impulsivity Tertile 2	1.67 (1.17–2.17)	
	CFC Tertile 3	1.67 (1.00–2.17)		Impulsivity Tertile 3	1.83 (1.17–2.50)	

Emotional eating score ranges from 1 to 4

*CFC-12* Consideration of Future Consequences scale, high CFC-12 scores indicate a high level of future orientation

*BIS-11* Barratt Impulsiveness Scale (11th version), high BIS-11 scores indicate a high level of impulsivity

The three categories of CFC-12 and BIS-11 were calculated according to tertiles of the total score

^1^*p*-values are based on Kruskal-Wallis tests (corrected for multiple testing with a Holm-Bonferroni procedure)

### Association between EmE and repeated measures of BMI according to gender, CFC levels, and impulsivity levels

Tables 3 and 4 show the associations between EmE and repeated measures of BMI according to gender, as well as CFC and impulsivity. EmE was significantly associated with BMI in every tertiles of CFC or impulsivity, and in both men and women. Associations were stronger in women than in men. Even though CFC and impulsivity did not moderate the effect of EmE on change of BMI (non-significant second-order interaction tests), these constructs moderated the effect of EmE on BMI. The association between EmE and BMI was stronger for participants with a low level of CFC (Tertile 1) compared with participants with an average level of CFC (Tertile 2) and with a high level of CFC (Tertile 3) in both women and men. Tertile 2 and Tertile 3 were also different in women, whereas no difference was found in men (see Additional files 1 and 2). In addition, the association between EmE and BMI was stronger for women with a high level of impulsivity (Tertile 3) compared to women with an average level of impulsivity (Tertile 2) and a low level of impulsivity (Tertile 1) (see Additional file 3). No difference was found between Tertile 1 and Tertile 2. Impulsivity did not moderate the association between EmE and BMI among men.

**Table 3:** Association between emotional eating and repeated measures of BMI according to consideration of future consequences categories (CFC) (NutriNet-Santé study, 2014)

Gender	CFC	EmE β^1^ (95% CI)	P^2^	EmE × CFC-12 β^3^ (95% CI)	P^2^	EmE × CFC-12 β^3^ (95% CI)	P^2^
Women	CFC Tertile 1	1.72 (1.63, 1.81)	<.0001	Ref			
	CFC Tertile 2	1.48 (1.37, 1.59)	<.0001	−0.23 (− 0.37, − 0.09)	.007	Ref	
	CFC Tertile 3	1.29 (1.20, 1.38)	<.0001	−0.43 (− 0.55, − 0.30)	<.0001	−0.19 (− 0.33, − 0.05)	.03
Men	CFC Tertile 1	1.18 (1.04, 1.33)	<.0001	Ref			
	CFC Tertile 2	0.83 (0.65, 1.00)	<.0001	−0.36 (−0.58, − 0.13)	.01	Ref	
	CFC Tertile 3	0.91 (0.76, 1.06)	<.0001	−0.27 (− 0.48, − 0.07)	.03	0.08 (− 0.15, 0.31)	.48

*CFC-12* Consideration of Future Consequences scale, high CFC-12 scores indicate a high level of future orientation

The three categories of CFC-12 were calculated according to tertiles of the total score

EmE, Emotional Eating, is continuous variable ranging from 1 to 4

^1^β coefficients of the EmE effect can be interpreted as changes in BMI (in kg/m^2^) per increase of 1 point in the EmE scale in each CFC category

^2^Adjusted p-value (correction for multiple testing with a Holm-Bonferroni procedure) based on linear mixed-effects models with time, age, education level, occupational status, monthly income household unit, smoking status, physical activity, and history of dieting as fixed effects, and intercept and time as random effects

^*3*^β coefficients of the EmE × CFC-12 interaction can be interpreted as differences of BMI slope (in kg/m^2^) per increase of 1 point in the EmE scale between CFC categories

**Table 4:** Association between emotional eating and repeated measures of BMI according to impulsivity categories (NutriNet-Santé study, 2014)

Gender	BIS-11	EmE β^1^ (95% CI)	P^2^	EmE × BIS-11 β^3^ (95% CI)	P^2^	EmE × BIS-11 β^3^ (95% CI)	P^2^
Women	Impulsivity Tertile 1	1.35 (1.25, 1.46)	<.0001	Ref			
	Impulsivity Tertile 2	1.47 (1.38, 1.57)	<.0001	0.12 (−0.02, 0.26)	.18	Ref	
	Impulsivity Tertile 3	1.73 (1.63, 1.82)	<.0001	0.37 (0.24, 0.51)	<.0001	0.26 (0.12, 0.39)	.0009
Men	All	1.00 (0.91, 1.10)	<.0001	–		–	

*BIS-11*, Barratt Impulsiveness Scale (11th version), high BIS-11 scores indicate a high level of impulsivity

The three categories of BIS-11 were calculated according to tertiles of the total score

EmE, Emotional Eating, is continuous variable ranging from 1 to 4

^1^β coefficients of the EmE effect can be interpreted as changes in BMI (in kg/m^2^) per increase of 1 point in the EmE scale in each impulsivity category

^2^Adjusted p-value (correction for multiple testing with a Holm-Bonferroni procedure) based on linear mixed-effects models with time, age, education level, occupational status, monthly income household unit, smoking status, physical activity, and history of dieting as fixed effects, and intercept and time as random effects

^3^β coefficients of the EmE × BIS-11 interaction can be interpreted as differences in change of BMI (in kg/m^2^) per increase of 1 point in the EmE scale between impulsivity categories

## Discussion

As previously observed in the literature, EmE was positively associated with BMI in men and women, with the latter showing a stronger relationship. CFC and impulsivity quantitatively moderated the association between EmE and BMI, but did not influence the association between EmE and BMI change. The association between EmE and BMI was stronger in women and men with a low future orientation, and in women with a high level of impulsivity.

### Characteristics of emotional eaters according to CFC and impulsivity

Although less future oriented women (corresponding to a lower CFC) had a higher EmE score, this association was marginal and no association was observed in men. These data suggest that concerns with future consequences is not correlated with EmE, even though data in the literature indicate that higher level of CFC was associated with healthier behavior such as healthy eating, more exercise, and smoking cessation [60]. As shown by previous work, the depletion of self-regulatory resources and conflicting goals can reduce the ability to regulate one’s behavior [61], which could therefore increase desire for immediate rewards. Another study showed that a higher level of immediate concerns was a better predictor of low self-control compared to a higher level of future concerns [50].

In agreement with the literature, EmE and impulsivity were positively associated in both men and women [62–64]. A high level of impulsivity could lead to impairments in inhibitory control which impede impulsive individuals to resist urges to eat, especially in response to negative emotional states [63]. The inhibition (of urge to eat) could be manifested by delaying, suppressing, or completely preventing the impulsive action [34].

### EmE and BMI change

In agreement with cross-sectional [7, 19–22] and longitudinal studies [22–24], EmE was positively associated with BMI in our sample. Our results showed a stronger relationship between EmE and BMI in women, which is consistent with some studies [17, 20, 21], but not all [19, 65]. More specifically, EmE was also positively associated with an increase in BMI over the period of follow-up (data not shown). However, this effect was not moderated by CFC and impulsivity. This absence of moderation could be related to a lack of statistical power regarding second-order interaction tests [66], particularly among men. It is also possible that the duration of the follow-up was too short to observe a meaningful difference. Moreover, we did not obtain repeated measures of CFC and impulsivity to assess individual changes regarding these scales.

### Influence of CFC on the association between EmE and weight status

CFC moderated the relationship between EmE and weight status in women and men. The more future oriented the individuals were, the weaker was the relationship between EmE and BMI, indicating the synergism between CFC and EmE. In the literature, CFC was shown to be associated negatively with BMI [31] and positively with several behavioral outcomes, such as healthy eating, lower smoking, and a decreased consumption of alcohol [60]. Our results could indicate a protective effect of CFC in dealing with negative emotions. Considering potential negative consequences of one’s behavior such as weight gain could be a mean that individuals use when feeling urges to eat in response to negative emotions. However, positive outcomes could be a more effective solution to regulate immediate temptations [67, 68]. Individuals with high CFC may as a consequence eat smaller portions of foods or select food with lower energy content. Individuals with a more pronounced future perspective measured by the Zimbardo Time Perspective Inventory also reported to be more mindful [69] (i.e having nonjudgmental attention, openness, and an acceptance of immediate experience [70]), and these constructs are thought to share common mechanisms [71, 72]. In mindfulness-based interventions, individuals are trained to attend to negative feelings and accept them, which allow a better consideration of potential consequences of one’s behavior on the body. Consequently, mindfulness has been shown to increase self-regulatory resources [73], which could lead to lower concerns over immediate temptations. These interventions have been found successful in reducing EmE occurrences and diminishing the urge to emotionally overeat [74, 75]. Another strategy based on episodic future thinking, “a projection of the self into the future to pre-experience an event” [76], found a reduction of snacking (as well as caloric intake coming from snacks) and diminished the need for immediate gratification [77].

### Influence of impulsivity on the association between EmE and weight status

Impulsivity moderated the relationship between EmE and BMI in women. The more impulsive the individuals were, the stronger was the relationship between EmE and BMI. To our knowledge, the potential moderation effect of impulsivity on the relationship between EmE and weight status has not been previously studied. An impulsive behavior was found associated with weight status [3, 35, 46–49], a tendance to overeat [35–37], and eating disorders [38–44]. Highly impulsive individuals are more susceptible to present immediate impulses to eat in response to negative emotional states [62, 78, 79] while less impulsive individuals show a greater mindfulness [69]. Impulsivity may lead individuals with EmE to have more eating episodes following emotions, or to experience episodes of greater intensity, with intake of larger portions. A better inhibitory control could lead to fewer inner impulses and lower the tendency to eat an excessive amount of food, as shown by studies using inhibitory control trainings [80–82].

### Gender differences

Marginal significant gender differences were found in our study concerning the level of CFC and impulsivity. In the literature, no gender differences were found regarding the level of CFC [32], and mixed findings were found regarding differences in impulsivity [51, 83]. CFC and impulsivity moderated the relationship between EmE and BMI mostly in women. Moderating effects in men were less consistent. The psychological determinants influencing the ability to control urges to eat in response to negative emotions could therefore be different among impulsive men. These results illustrate the need to take gender into account when studying associations between eating behavior and health markers such as weight status.

### Strengths and limitations

The main strength of this study is its large sample size with individuals of various socio-demographic characteristics and nutritional status, which allows the use of multiple covariates to adjust for confounding factors. However, we cannot rule out the possibility that other important confounders were not taken into account such as levels of anxiety or stress. To our knowledge, only a few studies have assessed the moderating effect of psychological traits in the relationship between EmE and weight status. Thus, this study offers novel perspectives to better understand EmE and its association with weight. One limitation of our study concerns the delay between the administration of the TFEQ-R21 and the CFC-12 and BIS-11. Changes in EmE, CFC and impulsivity for some participants could have occurred during this period. However, as they represent psychological constructs, a low time variability can be assumed, as it has been suggested for impulsivity [51]. Another limit is the self-reported anthropometric measures, which could have led to attribution errors. However, standardized clinical measurements on a subsample (*n* = 2513) of the NutriNet-Santé cohort showed a good convergence with self-reported data [84]. Our study could also present a selection bias because of the method used to recruit participants, which was based on volunteering. Consequently, our subjects may have high health awareness compared to the global population, and may not be representative of the French population. Recent studies reported a two-factor structure of the CFC-12, distinguishing immediate and future subscales. Even though there is no consensus on the use of the CFC-12, a two-dimension analysis could have added another perspective in our interpretation of the results. The BIS-11 questionnaire is one of the most used self-report measures of impulsivity. However, considering the multidimensionality of impulsivity as a construct and its various methods of measurement, caution is needed when interpreting and extrapolating our results.

## Conclusions

In conclusion, our results show that CFC moderates the association between emotional eating and BMI (but not BMI change) among men and women. We also observed a similar moderation effect of impulsivity on the association between emotional eating and BMI (but not BMI change) in women. Being more future oriented could increase emotional self-control, and being less impulsive could help to reduce the occurrence of urges to eat. CFC and impulsivity could therefore represent two important psychological traits to take into account when attempting to prevent overeating in response to negative emotions. Interventional strategies targeting cognitive control such as inhibitory control training, episodic future thinking, message framing, and self-awareness (e.g., mindfulness based interventions) could be tested for their ability to increase consideration of future consequences and/or decrease impulsivity in relation to eating behavior.

## Additional files

Additional file 1: Association between emotional eating and BMI according to consideration of future consequences categories (CFC) in women. (DOCX 19 kb)

Additional file 2: Association between emotional eating and BMI according to consideration of future consequences categories (CFC) in men. (DOCX 20 kb)

Additional file 3: Association between emotional eating and BMI according to impulsivity categories in women. (DOCX 19 kb)

## Abbreviations

BIS-11: Barratt Impulsiveness Scale
CFC: Consideration of future consequences
CFC-12: Consideration of future consequences scale
CNIL: Commission Nationale Informatique et Libertés
CU: Consumption unit
EmE: Emotional eating
IRB: International Research Board of the French Institute for Health and Medical Research
TFEQ-R21: Revised 21-item Three-Factor Eating Questionnaire
