# Supplementary figures and images for: Impulsivity and consideration of future consequences as moderators of the association between emotional eating and body weight status
Source: Int J Behav Nutr Phys Act. 2018 Sep 6;15:84. doi: 10.1186/s12966-018-0721-1 (PMC6127957; doi:10.1186/s12966-018-0721-1)

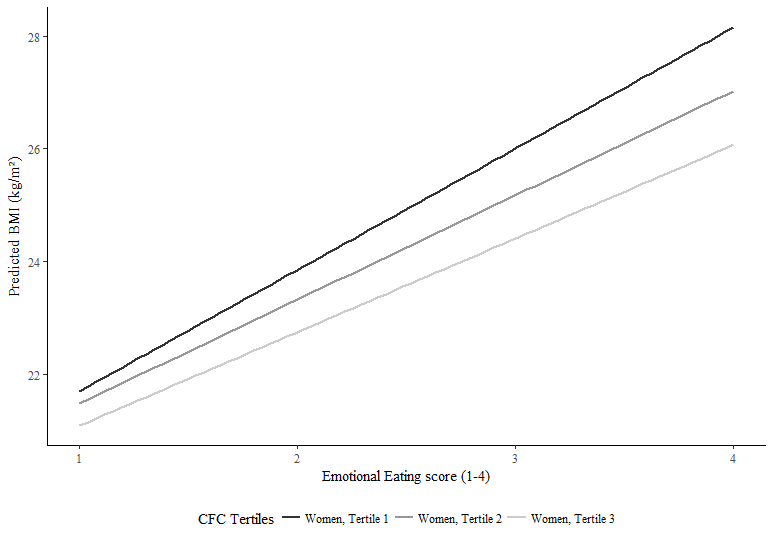

Supplement: Supplementary file 1 — Association between emotional eating and BMI according to consideration of future consequences categories (CFC) in women. (DOCX 19 kb) [file 12966_2018_721_MOESM1_ESM.docx]

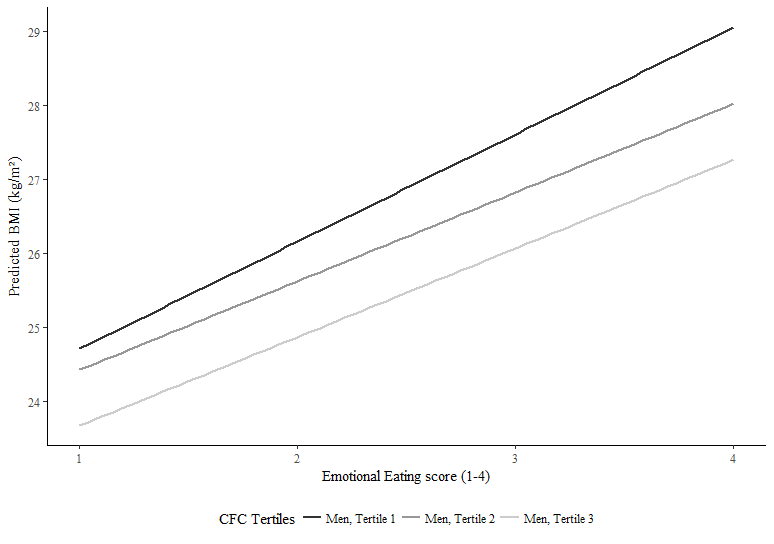

Supplement: Supplementary file 2 — Association between emotional eating and BMI according to consideration of future consequences categories (CFC) in men. (DOCX 20 kb) [file 12966_2018_721_MOESM2_ESM.docx]

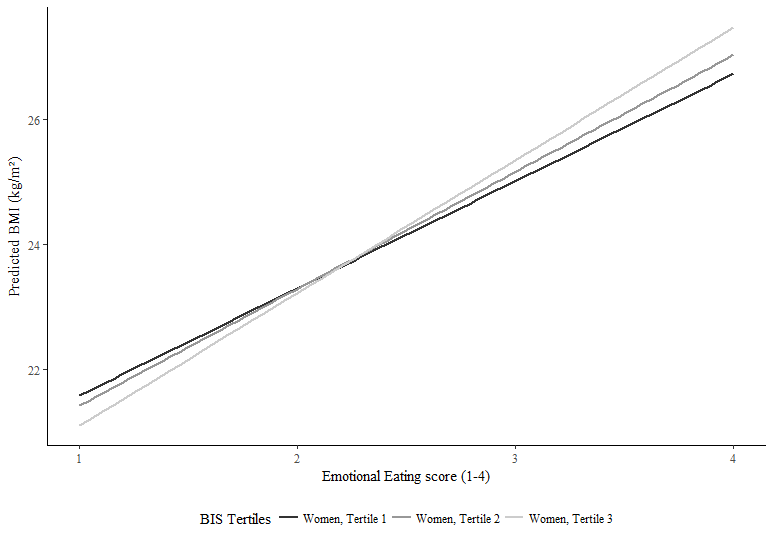

Supplement: Supplementary file 3 — Association between emotional eating and BMI according to impulsivity categories in women. (DOCX 19 kb) [file 12966_2018_721_MOESM3_ESM.docx]
